# Computational and Enzymatic Studies of Sartans in SARS-CoV-2 Spike RBD-ACE2 Binding: The Role of Tetrazole and Perspectives as Antihypertensive and COVID-19 Therapeutics

**DOI:** 10.3390/ijms24098454

**Published:** 2023-05-08

**Authors:** Konstantinos Kelaidonis, Irene Ligielli, Spiros Letsios, Veroniki P. Vidali, Thomas Mavromoustakos, Niki Vassilaki, Graham J. Moore, Weronika Hoffmann, Katarzyna Węgrzyn, Harry Ridgway, Christos T. Chasapis, John M. Matsoukas

**Affiliations:** 1 NewDrug PC, Patras Science Park, 26504 Patras, Greece; k.kelaidonis@gmail.com (K.K.); lespir2@gmail.com (S.L.); 2 Department of Chemistry, Laboratory of Organic Chemistry, National Kapodistrian University of Athens, 15772 Athens, Greece; eir.ligielli@gmail.com (I.L.);; 3 Natural Products and Bioorganic Chemistry Laboratory, Institute of Nanoscience & Nanotechnology, NCSR “Demokritos”, 15341 Athens, Greece; v.vidali@inn.demokritos.gr; 4 Laboratory of Molecular Virology, Hellenic Pasteur Institute, 11521 Athens, Greece; nikiv@pasteur.gr; 5 Department of Physiology and Pharmacology, Cumming School of Medicine, University of Calgary, Calgary, AB T2N 4N1, Canada; mooregj@shaw.ca; 6 Laboratory of Virus Molecular Biology, Intercollegiate Faculty of Biotechnology of University of Gdańsk and Medical University of Gdańsk, University of Gdańsk, Abrahama 58, 80-307 Gdansk, Poland; weronika.hoffmann@biotech.ug.edu.pl; 7 Laboratory of Molecular Biology, Intercollegiate Faculty of Biotechnology of University of Gdańsk and Medical University of Gdańsk, University of Gdańsk, Abrahama 58, 80-307 Gdansk, Poland; katarzyna.wegrzyn@biotech.ug.edu.pl; 8 Institute for Sustainable Industries and Liveable Cities, Victoria University, Melbourne, VIC 8001, Australia; 9 AquaMem Consultants, Rodeo, NM 88056, USA; 10 Institute of Chemical Biology, National Hellenic Research Foundation, 11635 Athens, Greece; 11 Institute for Health and Sport, Victoria University, Melbourne, VIC 3030, Australia; 12 Department of Chemistry, University of Patras, 26504 Patras, Greece

**Keywords:** SARS-CoV-2, COVID-19, bisartans, tetrazole, ACE2, RAS, RBD

## Abstract

This study is an extension of current research into a novel class of synthetic antihypertensive drugs referred to as “bisartans”, which are bis-alkylated imidazole derivatives bearing two symmetric anionic biphenyltetrazoles. Research to date indicates that bisartans are superior to commercially available hypertension drugs, since the former undergo stronger docking to angiotensin-converting enzyme 2 (ACE2). ACE2 is the key receptor involved in SARS-CoV-2 entry, thus initiating COVID-19 infection and in regulating levels of vasoactive peptides such as angiotensin II and beneficial heptapeptides A(1-7) and Alamandine in the renin–angiotensin system (RAS). In previous studies using in vivo rabbit-iliac arterial models, we showed that Na^+^ or K^+^ salts of selected Bisartans initiate a potent dose–response inhibition of vasoconstriction. Furthermore, computational studies revealed that bisartans undergo stable binding to the vital interfacial region between ACE2 and the SARS-CoV-2 “receptor binding domain” (i.e., the viral RBD). Thus, bisartan homologs are expected to interfere with SARS-CoV-2 infection and/or suppress disease expression in humans. The primary goal of this study was to investigate the role of tetrazole in binding and the network of amino acids of SARS-CoV-2 Spike RBD-ACE2 complex involved in interactions with sartans. This study would, furthermore, allow the expansion of the synthetic space to create a diverse suite of new bisartans in conjunction with detailed computational and in vitro antiviral studies. A critical role for tetrazole was uncovered in this study, shedding light on the vital importance of this group in the binding of sartans and bisartans to the ACE2/Spike complex. The in silico data predicting an interaction of tetrazole-containing sartans with ACE2 were experimentally validated by the results of surface plasmon resonance (SPR) analyses performed with a recombinant human ACE2 protein.

## 1. Introduction

Since the advent of the Severe Acute Respiratory Syndrome Coronavirus (SARS-CoV-2) pandemic in late 2019, intense research into the discovery and design of precision antiviral drugs was conducted [1,2,3,4,5,6,7,8,9]. The recent announcement of the Pfizer and Moderna antivirals, Paxlovid and Molnupiravir, respectively, are examples of ongoing research and development efforts in this area [10,11,12,13,14,15]. The drugs have different mechanisms of action, with Paxlovid inhibiting the main 3CL viral protease and Molnupiravir interfering with RNA-dependent RNA polymerase. Such antivirals are crucial armaments in fighting not only the current pandemic but also future outbreaks, since they are less expensive to manufacture and distribute than vaccine therapies, and they can be orally administered in a prophylactic regimen or post-infection to minimize severe symptoms and hospitalizations. Furthermore, the combination of drugs operating through different mechanisms may optimize the inhibitory effect through synergy [16]. Similar therapeutic approaches were applied to other viruses, such as the human immunodeficiency virus [17,18].

**Figure 1:**
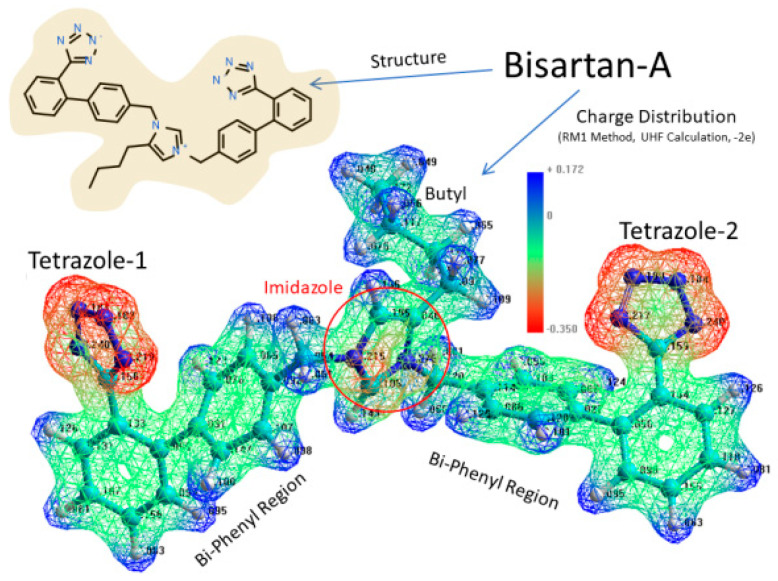
Structure and charge distribution of Bisartan-A, a bis-alkylated imidazole biphenyltetrazole at pH 7.4. The structure of BisA is illustrated in the upper left panel and electrostatic charge distribution in the lower image of Bisartan-A. Negatively charged regions = red; neutral to positive regions = green to blue. Graph by Ridgway et al. [22].

**Figure 2:**
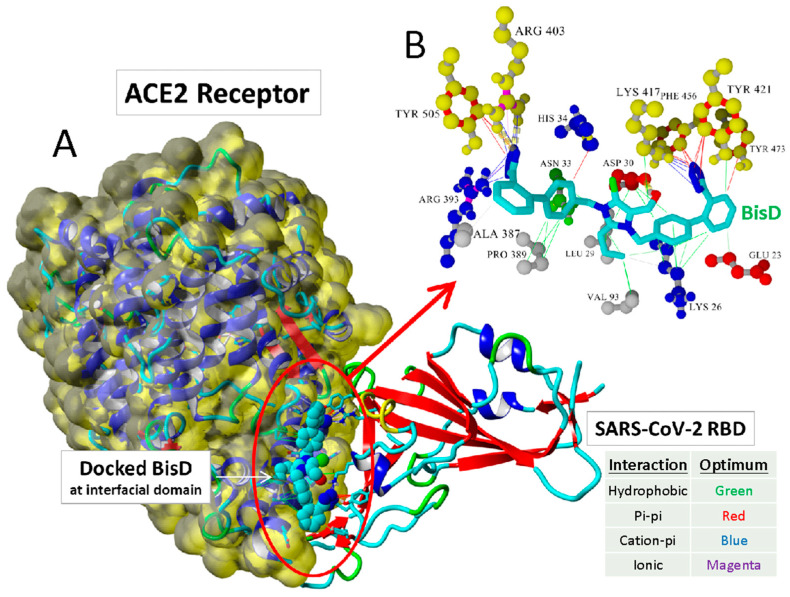
(**A**) Docking of Bisartan-D (bearing the butyl group at position 2 of imidazole and two symmetric N,N′ biphenyl tetrazoles) and of a chlorinated bisartan, BisC (bisalkylated losartan), to the critical interfacial region between the ACE2 receptor (Van der Waals surface) and the SARS-CoV-2/RBD (PDB ID: 6LZG). (**B**) The BisC binding motif involved pi/pi (red lines), pi/cation (blue lines), and hydrogen bonding (thick dashed lines) interactions with the SARS-CoV-2 RBD residues (yellow spheres). The binding of BisC to the ACE2 interfacial region was dominated by hydrophobic interactions (green lines) and secondarily by pi–cation interactions (blue lines) to Arg393. Graph by Ridgway et al. [22].

**Figure 3:**
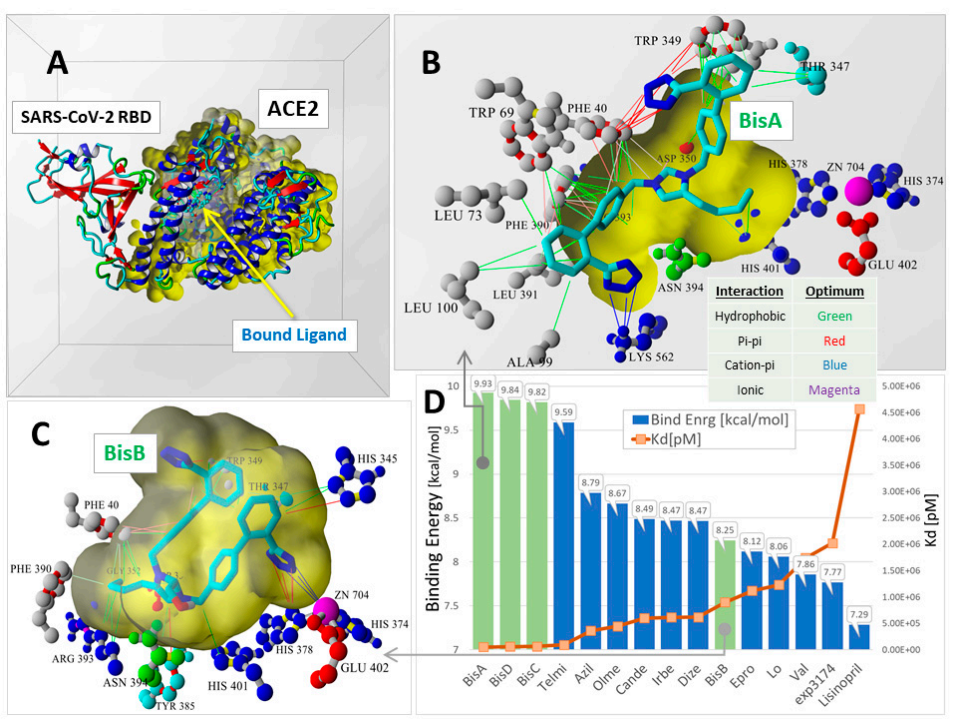
Global docking of 15 ARBs to the 6LZG ACE2-SARS-CoV-2 RBD complex by Ridgway et al. [22]. Docking was performed using AutoDock VINA (YAMBER3 force field) with 100 runs for each ARB. The docking domain comprised a rectilinear cell with non-periodic (wall) boundaries 8 Å from any target atom. (**A**) Overview of 6LZG showing the major ACE2 channel where ARBs preferentially underwent docking. (**B**) The binding motif of bisartan BisA involved hydrophobic (green lines), pi–pi-resonance (red lines), and cation–pi (blue lines) interactions. Although the BisA conformational pose shown was proximate to the Zn^2+^ cofactor and also exhibited the strongest binding (9.93 kcal/mol) compared to all other ARBs, it did not directly coordinate with the Zn^2+^ cofactor. (**C**) The binding motif of BisB (8.25 kcal/mol) underwent coordination with Zn^2+^ via pi–pi resonance (red lines) and cation-pi (blue lines) interactions. (**D**) VINA docking scores (bars) and dissociation constants (Kd; orange line) were calculated for the best poses of all 15 ARBs. With the exception of BisB, the bisartans (green-shaded bars) consistently exhibited stronger ACE2 binding compared to all other ARBs.

Our research team discovered and synthesized a new cohort of antihypertensive drugs that were predicted to stably dock at catalytic site and semi-stably at furin-cleavage site (residues 681–686) of the transmembrane angiotensin-converting enzyme 2 (ACE2), which is the primary receptor for attachment and entry of SARS-CoV-2 into susceptible host tissues [19,20,21,22,23,24]. We refer to this new class of drugs as “bisartans” since they share structural similarities to the “sartan” therapeutics (e.g., Losartan, Olmesartan, Telmisartan, Irbersartan, Valsartan, etc.) that currently dominate the global market for the treatment of hypertension and heart disease. Bisartans are bis-alkylated imidazole derivatives bearing dual symmetric anionic biphenyltetrazole moieties (Figure 1) [22]. They exhibit a proclivity to interact with metallo-receptors/enzymes, particularly those harboring Zn^2+^ cofactors. In general, the zinc-binding motif in metalloenzymes or metalloproteases is rich in histidines [25], which are excellent sources of pi–pi interactions with ligands containing phenyl groups such as sartans. Our computational modelling and in vivo experimental results (from rabbit-iliac arterial models) suggest that bisartans could be beneficial for the treatment of not only heart disease, diabetes, renal dysfunction, and related vascular illnesses, but also COVID-19. The in silico docking and molecular dynamics (MD) simulations revealed that several bisartan homologs (such as BisA, BisB, BisC, and BisD) [22] exhibited enhanced binding affinity for the ACE2/Spike protein complex (PDB ID: 6LZG) compared to other known (marketed) sartans, including the angiotensin receptor I blocker lisinopril (Figure 2 and Figure 3) [22]. Bisartans undergo stable docking to the Zn^2+^-domain of the ACE2 catalytic site, as well as the critical interfacial region between ACE2 and the SARS-CoV-2 receptor-binding domain (Figure 3). Additionally, bisartans exhibit semi-stable docking to the furin-cleavage site (residues 681–686) of the SARS-CoV-2 Spike protein required for viral entry into host cells. The docking studies suggest furin inhibition could occur by drug interaction with this arginine-rich site and, in particular, the P681R mutation which conveys enhanced infectivity to the Delta variant [19]. These findings strongly point to the potential of bisartans to function as a novel class of antiviral agents in addition to their antihypertensive action [21,22].

The major goal of our research was to further develop and experimentally evaluate in vitro and in vivo the efficacy of bisartans, not only as multifunctional SARS-CoV-2 antiviral agents but also as antihypertension and antibacterial drugs. In addition, we plan to design a range of bisartan structural analogs and/or pharmacophore modifications and computationally evaluate their behavior against additional human disease targets (receptors and enzymes), especially those containing metal cofactors. To achieve these objectives, we plan to focus on the power of the latest machine-learning chem/bioinformatics approaches to drug discovery and design, including the implementation of advanced algorithms offered by Molsoft, Schroedinger, and other commercial and open-source software developers. In addition, by the use of surface plasmon resonance technology, we aim to experimentally validate the binding capacity of sartans to the SARS-CoV-2 receptor ACE2.

## 2. Results

### 2.1. Computational Studies on the Interactions of Ligands with Spike Protein/ACE2

The effect of our generated ARBs and some well-known sartans on the SARS-CoV-2 spike protein/ACE2 region was investigated using docking and PLIP (PDB ID: 6LZG) [10.1016/j.cell.2020.03.045], and their binding affinity is shown at Table 1. The smaller molecules **Co1** and **Co2** failed to bind effectively and provided almost the same score, with the active site of the S-protein due to the limited number of interactions. This can be also excused due to the lack of aromatic rings that can provide the molecule to bind with the protein with π–stacking and π-–cation interactions. Both molecules formed hydrogen bonds with 353A and 496B Gly, as well as hydrophobic bonds with 497B Phe and 505B Tyr. In an effort to form interactions with aromatic rings, we decided to keep the trt groups in our molecules. **Co3** and **Co5** displayed a higher binding affinity of −9.4 and −9.71 kcal/mol, respectively. Compared to valsartan, olmesartan, eprosartan, and losartan (−8.53, −7.88, −6.98 and −7.74 kcal/mol), these molecules bound more effectively (Table 1 and Table 2). Those molecules formed the biggest number of bonds compared to the other compounds due to their aromaticity. Although they did not form the same bonds with the S-protein, they formed similar ones. They both formed hydrophobic interactions with 505B Tyr, a π–stacking with 34A His, and a π–cation interaction with 403B Arg. Following the same pattern, we decided to generate **Co7** and **Co9** with the only difference from the previous group being the presence of a -CH_2_OH group, we thought that maybe more hydrogen bonds can be formed. However, that did not happen; on the contrary, the new compounds had a lower binding affinity (−6.8 and 6.81 kcal/mol) compared to **Co3** and **Co5**. Again, all the compounds were aimed at the active site of S-protein; thus, all of them interacted with the same amino acids. **Co7** and **Co9** formed hydrogen bonds with 33A Asn and 393A Arg, hydrophobic interactions with 30A Asp, 33A Asn, 34A His, 37A Glu, 387A Ala, and 417B Lys. The interactions are shown in detail with their respective distances in Table 3.

Then, we removed the -trt group from the same molecules. As shown, by removing that group, the binding affinity of those molecules increased significantly in all the cases, with the most drastic increase in the case of **Co9** and **Co10,** making the latter an excellent ligand (−11.4 kcal/mol). By removing the -trt from our previously best-performing molecule, **Co5**, the produced **Co6** had an even lower binding energy (−12.1 kcal/mol). The resulting **Co4** and **Co8** also bound strongly to S-protein. By decreasing the size of the previous molecules, thus limiting their hydrophobic nature, we observed fewer hydrophobic bonding. However, it was also apparent that the number and quality of the forming hydrogen bonds increased. This was due to the fact that the -trt groups can limit the compounds’ ability to be near several amino acids due to the steric effect, which, otherwise, could bind with them effectively. The interactions are shown in detail with their respective distances in Table 4.

**Figure 4:**
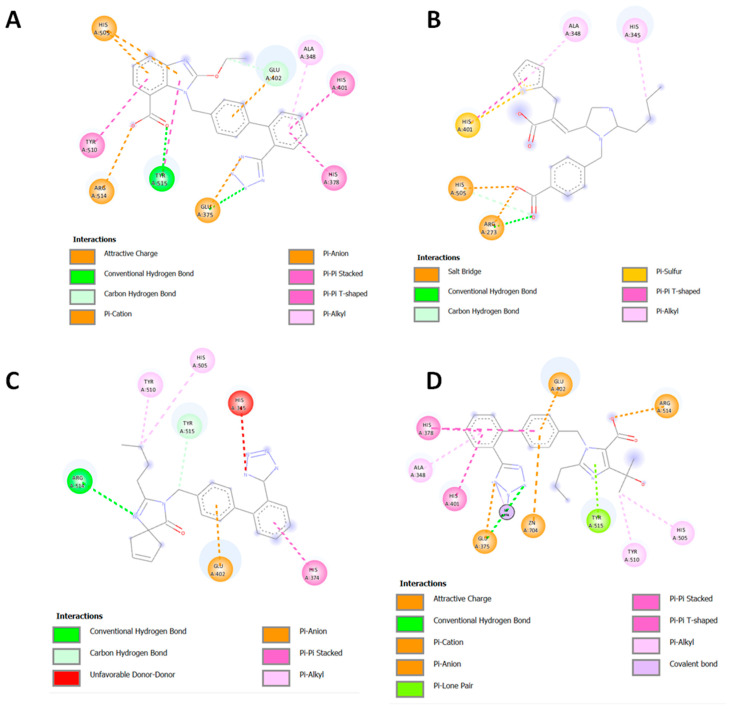
Interactions of candesartan (**A**), eprosartan (**B**), irbesartan (**C**), and olmesartan (**D**) with ACE2 of ACE2/Spike protein complex.

To elucidate the binding abilities of the new class of sartans to the ACE2/Spike protein complex, several binding experiments were performed for both the well-known sartans and the novel bisartan homologues. In the case of candesartan, eprosartan, irbesartan, and olmesartan (Figure 4), the ligands bound to the catalytic center of ACE2 through strong pi–pi Interactions between the sartans’ phenyl groups and histidines of the zinc-binding motif, including His 408 and 378. Additionally, salt bridges between carboxyl moiety and His 505 and Arg 273 of ACE2 stabilized the binding pose of eprosartan in the binding pocket. Tyr 515 of ACE2 played a critical role in the binding event of sartans due to its ability to interact via hydrogen bonds with nitrogen rings of ligands. 

### 2.2. Enzymatic Studies Supporting the Interaction of Sartans with ACE2

**Figure 5:**
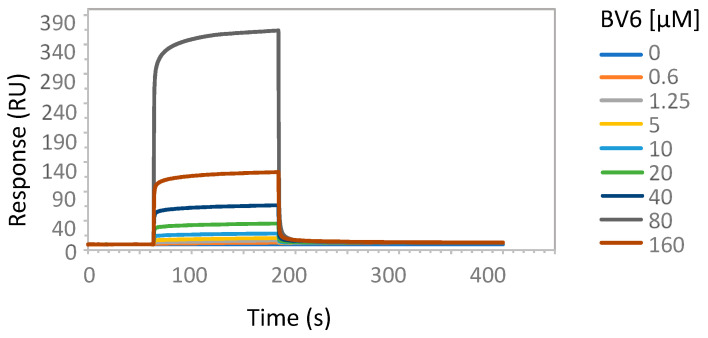
SPR analysis of BV6 interaction with ACE2 protein. The interaction of BV6 and ACE2 was analyzed with surface plasmon resonance, as described in Materials and Methods. The increasing amounts of BV6 (0.6, 1.25, 5, 10, 20, 40, 80, 160 µM) were run over the surface of a CM5 sensor chip with immobilized ACE2 protein.

**Figure 6:**
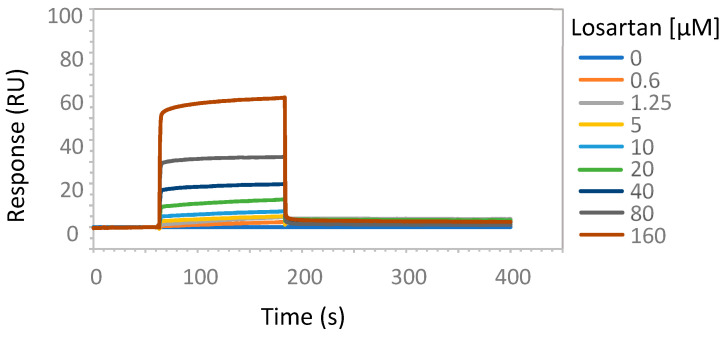
SPR analysis of losartan interaction with ACE2 protein. The interaction of losartan and ACE2 was analyzed with surface plasmon resonance, as described in Materials and Methods. The increasing amounts of losartan (0.6, 1.25, 5, 10, 20, 40, 80, 160 µM) were run over the surface of a CM5 sensor chip with immobilized ACE2 protein. The results are presented as sensorgrams obtained after subtracting the background response signal from a reference flow cell and a control experiment with buffer injection.

**Figure 7:**
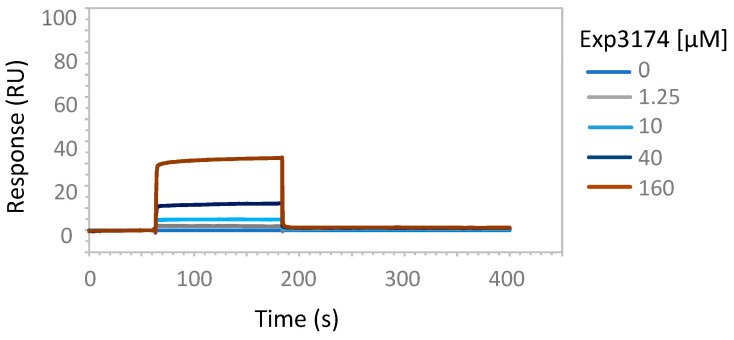
SPR analysis of Exp3174 interaction with ACE2 protein. The interaction of Exp3174 and ACE2 was analyzed with surface plasmon resonance, as described in Materials and Methods. The increasing amounts of Exp3174 (1.25, 10, 40, 160 µM) were run over the surface of a CM5 sensor chip with immobilized ACE2 protein.

To experimentally address the interaction of tetrazole-containing sartans with ACE2 protein, we performed SPR analysis using increasing amounts of the bisartan BV6 (containing two tetrazoles), the sartan losartan (containing one tetrazole and one hydroxyl) or its metabolite Exp3174 (losartan carboxylic acid containing one tetrazole and one carboxyl), and a recombinant human His-tagged ACE2 (Figure 5, Figure 6 and Figure 7). An efficient binding to ACE2 was shown for all sartans. The order of binding was BV6 > Losartan > Losartan carboxylic (Exp3174) and showed the superiority of tetrazolate compared to hydroxylate and carboxylate in binding to ACE2. For ACE2–Losartan complex formation, a kinetic constant was calculated with Biacore T200 Evaluation Software using the 1:1 binding model. The KD was 1.37 × 10^−8^ M (SE 1.5 × 10^−8^ M).

## 3. Discussion

### 3.1. Computational Approaches

Using state-of-the-art computational approaches, we identified a number of therapeutic targets for bisartans, namely three targets essential for viral infection and replication (i.e., ACE2, Furin, 3CLpro) [22]. These studies identified two additional therapeutic targets for bisartans, namely: (1) the zinc-dependent metallo-beta-lactamase from Bacillus cereus [20], and (2) Neprilysin, a zinc-dependent type II integral membrane peptidase belonging to the M13 family [20]. Beta-lactamases are responsible for imparting multiple antibiotic resistance to a range of human bacterial pathogens via hydrolysis of the beta-lactam ring of penicillin and its many derivative antibiotics, severely limiting therapeutic options. Neprilysin (NEP), on the other hand, is a regulatory enzyme that degrades beneficial vasoactive peptides, such as the atrial natriuretic peptide, the brain natriuretic peptide, bradykinin, adrenomedullin, and endothelin-15. Thus, the inhibition of NEP by bisartans is expected to lead to increased levels of these peptides. The combination of NEP suppression and angiotensin receptor inhibition (e.g., by bisartans) is known to be superior to the inhibition of either agent alone and leads to vasodilation and reduction in extracellular fluids via Na^+^ ion excretion.

### 3.2. Hypertension and COVID-19 Mechanisms Are Similar

Cardiovascular disease is related to COVID-19 in terms of mechanisms that trigger infection [26,27,28,29]. The release of the cytokine storm in patients with severe pneumonia is related to the over-expression of toxic angiotensin II in the renin-angiotensin system (RAS) [30,31]. The role of RAS in autoimmune inflammation, in the pathogenesis of major human diseases, and as a therapeutic target, was extensively investigated [32,33,34,35]. The alteration from pro-inflammatory to anti-inflammatory secretion in autoimmunity was reported [36]. Clinical studies advocate in favor of the beneficial effects of ARBs in blocking infection [26,27,31]. Hypertension gained popularity among researchers due to its over-representation among COVID-19 patients [37]. Studies reported that hypertension is the most common co-morbidity observed in patients affected by SARS-CoV-2 [38,39,40]. The mechanisms that link pre-existing hypertension and COVID-19 are related to endothelial dysfunction and RAS imbalance. The conventional RAS (ACE/Ang II/AT1R) axis activation in parallel with nonconventional (ACE2/Ang 1–7/Mas) axis down-regulation was proposed to be the underlying factor leading to severe COVID-19 outcome in hypertension [41,42]. The imbalance that favors the pro-inflammatory state is proposed to be the center of COVID-19 pathophysiological mechanisms [43].

### 3.3. Anionic Groups of ANGII and ARBs Interact with Positive Sites of AT1R and ACE2

**Figure 8:**
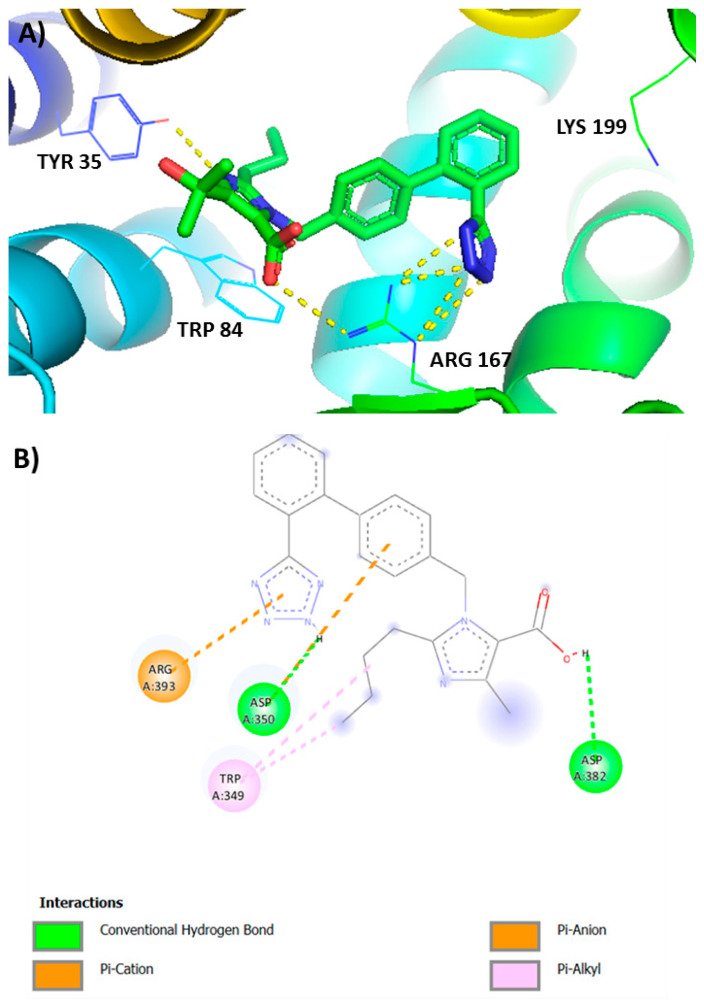
(**A**) Interactions of AT1R residues Lys 199, Arg 167, Trp 84, and Tyr 35 with olmesartan from crystal protein bank [28,29]. Arg167 of the AT1 receptor interacts with tetrazolate and carboxylate of olmesartan. (**B**) Interactions of Exp3174 (losartan carboxylic acid) and ACE2 in RBD spike-ACE2 complex. Tetrazole of Exp3174 interacts with ACE2 Arg393.

Recent crystal and in silico studies investigating the interactions between angiotensins and angiotensin receptor blockers (ARBs) with AT1R showed that sartans and bisartans which bear anionic tetrazolate and carboxylate groups as the pharmacophoric warheads bind to the 167Arg residue of AT1 receptor [28,29] and to the ACE2/RBD complex used by SARS-CoV-2 to infect cells [22]. In particular, olmesartan and carboxylic losartan (Exp3174) use the tetrazolate and carboxylate anionic groups to form the critical salt bridges with Arg 167 of AT1R and bisartans, bearing two tetrazolates, use both groups for the interaction with the receptor [19,21,22,44] (Figure 8). Aromatic interactions with Trp at position 84 strengthen the binding. As reported, the guanidino group of Ang II interacts with carboxyls of Asp363 and Asp282 of the AT1R and the anionic groups of the peptide are coordinated with Zn-bound ACE2. These studies showed the ability of bisartans to act as ARBs inhibiting vessel constriction in response to cumulative doses of ANGII and to reduce vasoconstriction due to the tight binding to Arg 167 of the AT1 receptor. The apparent targeting of multifunctional sites of the ACE2/RBD complex uncovered by in silico studies render sartans and bisartans bearing tetrazolate/carboxylate warheads promising drugs against COVID-19 [28,29,45,46].

### 3.4. Tetrazole of Sartans Interact with AT1R (Arg 167) and Spike 681–686 Arginines

Structure-activity, fluorescence, nuclear magnetic resonance, and computational studies led to a conformational model where the three aromatic amino acids (Tyr, His, Phe) formed a tripartite ring cluster with the hydroxylate of tyrosine to trigger activity [47,48,49,50,51,52,53]. The model and the importance of the three aromatic amino acids for activity were supported by the design and synthesis of appropriate constrained cyclic analogues [54] using Barlos 2-chlorotrityl resin [55]. Alterations in the three critical aromatic residues of angiotensin II deleted the agonist activity, confirming the importance of the aromatic residues for the activity of the peptide. A charge relay system was suggested to operate for triggering the activity of AT1R analogous to the charge relay system of serine proteases [56]. Anionic interactions between ANGII and AT1R were in analogy to the interactions between ARBs and AT1R identified in the crystal structure of the ARB olmesartan with AT1R [28,29]. The crystal structure shed light on the interactions and the critical role of the AT1R Arg167 bound to the two anionic pharmacophoric tetrazole and carboxylate groups of olmesartan. In silico studies showed that the ionic groups of sartans and bisartans, in particular tetrazole, bound to the positive Arg167 guanidino group of AT1R and the arginines of the ACE2/RBD complex [22]. These studies, furthermore, showed interactions of sartan tetrazole with arginines in the rich Arg cleavage site 681–686, thus preventing cleavage and infectivity [19,20,21,22].

### 3.5. Ligands Interacting with RBD/ACE2 Complex

**Figure 9:**
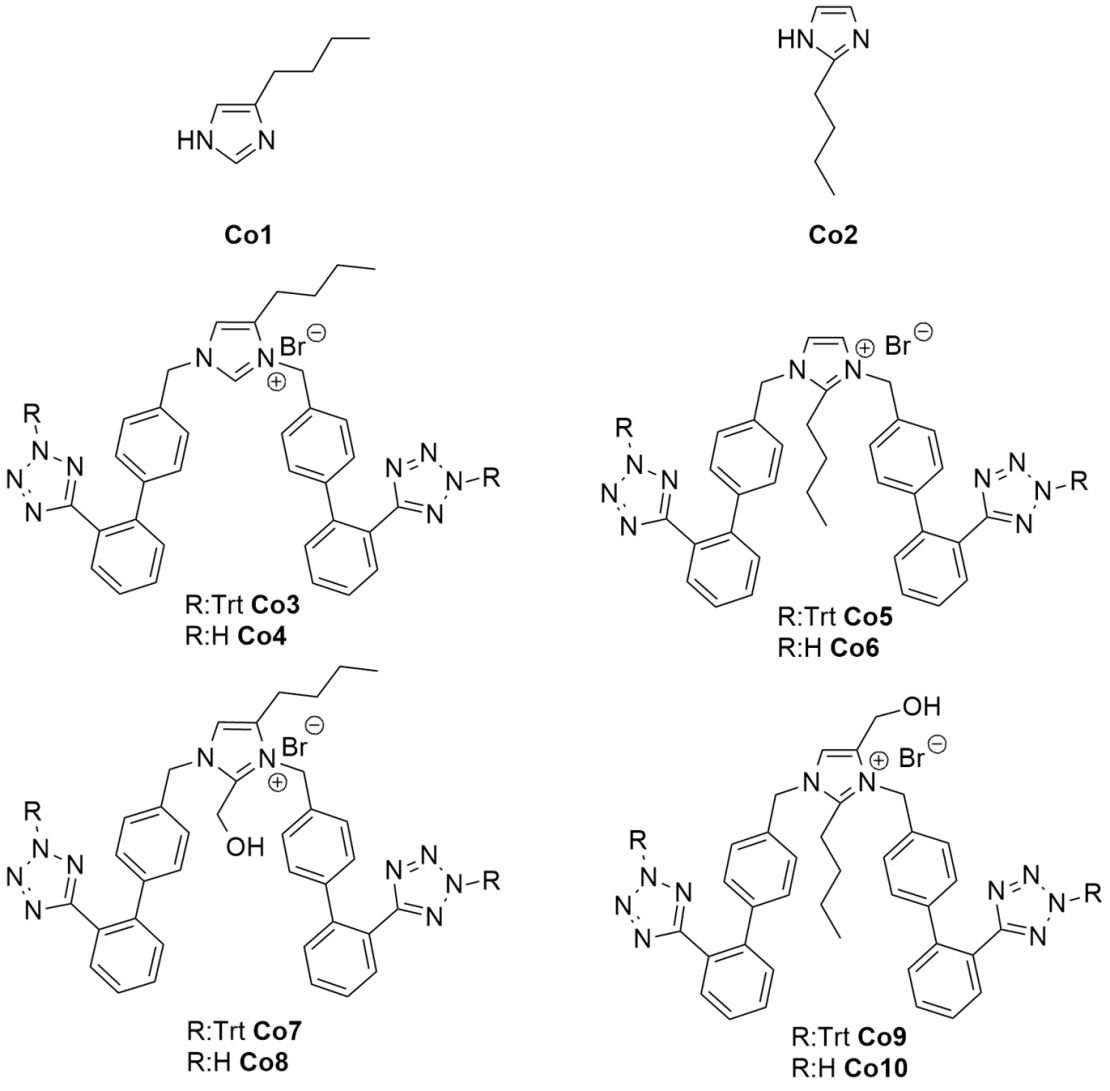
Structures of the used ligands. Bisartans are shown with protection of tetrazole with trityl group (**Co3**, **Co5**, **Co7**, **Co9**) and deprotected (**Co4**, **Co6**, **Co8**, **Co10**) (Scheme 1 shows the synthesis steps).

Figure 9 shows the structures of designed and synthesized imidazole-based ligands, protected by trityl group and unprotected, for studying their interaction with the RBD/ACE2 complex. Computational studies showed bisartan bisA (**Co4**) to be best docked with the RBD/ACE2 complex [22]. The free tetrazole of the investigated bisartans (**Co4**, **Co6**, **Co8**, **Co10**) bound stronger compared to trityl protected tetrazoles (**Co3**, **Co5**, **Co7**, **Co9**).

**Figure 10:**
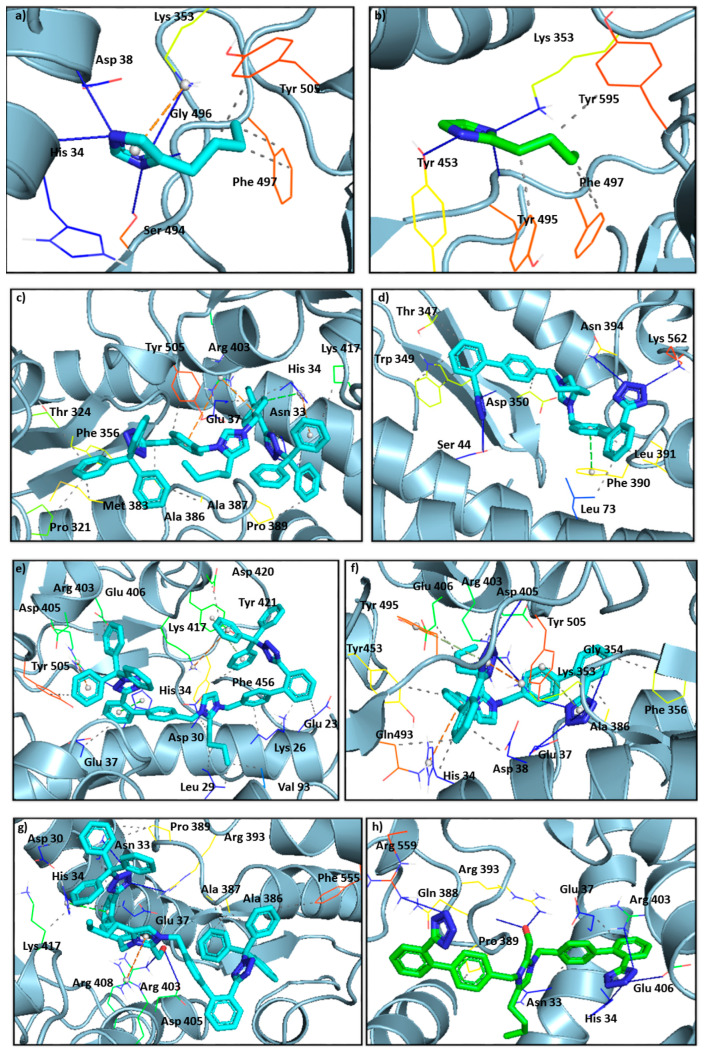

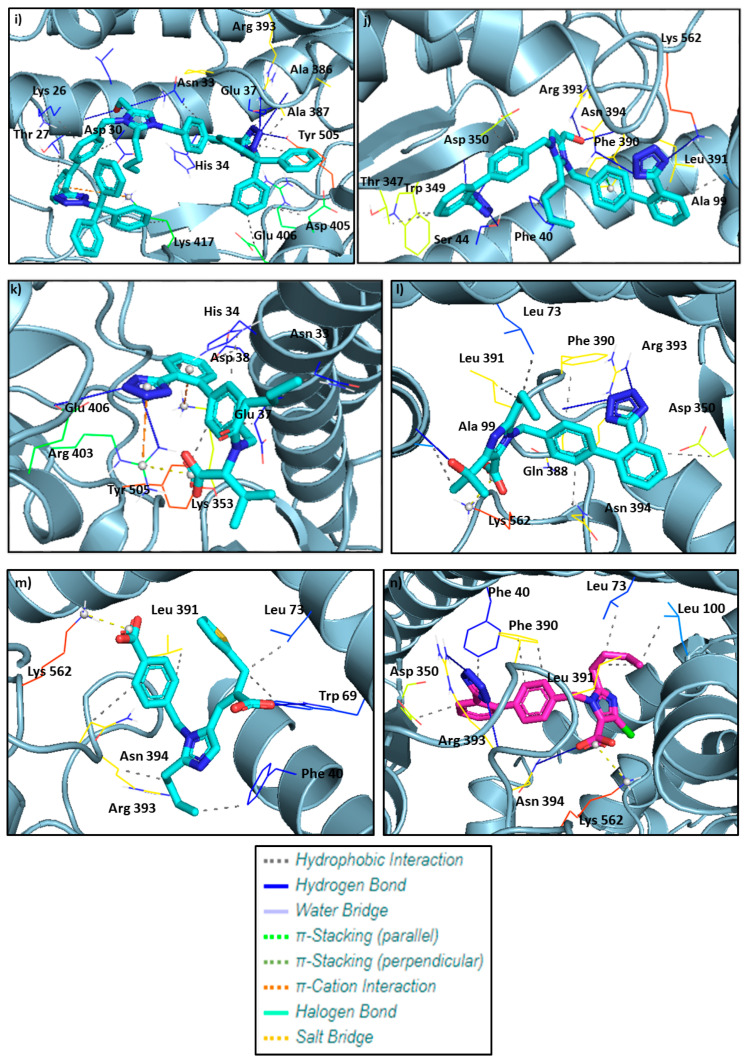
Crystallographic grids of the generated ligands (**Co1**–**Co10**), shown in (**a**–**j**), respectively, as well as the tested sartans (valsartan, olmesartan, eprosartan, losartan) in (**k**–**n**), incorporated into the cavity of the SARS-CoV-2 spike protein (PDB ID: 6LZG) of SARS-CoV-2 using a crystallographic grid. Of paramount importance is the hydrogen bonding of the tetrazolate with the amino acids. Namely **Co4** (**d**) forms hydrogen bonds with Ser 44, Asp 350, Asn 394, and Lys 562, **Co6** (**f**) with Glu 37, Lys 353, Gly 354, Arg 403, Gly 496, and Tyr 505, **Co8** (**h**) with His 34, Arg 403, Glu 406, and Arg 559, **Co10** (**j**) with Ser 44, Asp 350, Asn 394, and Lys 562.

**Figure 11:**
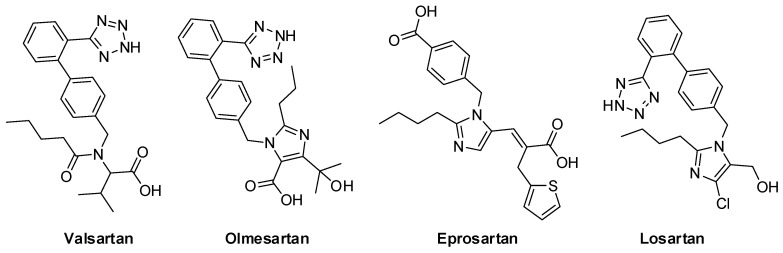
Chemical structures of sartans (valsartan olmesartan, eprosartan, and losartan) investigated in this study.

Figure 10 shows the crystallographic grids of the generated ligands (**Co1**–**Co10**) as well as the tested sartans valsartan, olmesartan, eprosartan, and losartan. The latter are depicted at Figure 11.

### 3.6. Imidazole and Benzimidazole Based Sartans

**Scheme 1:**
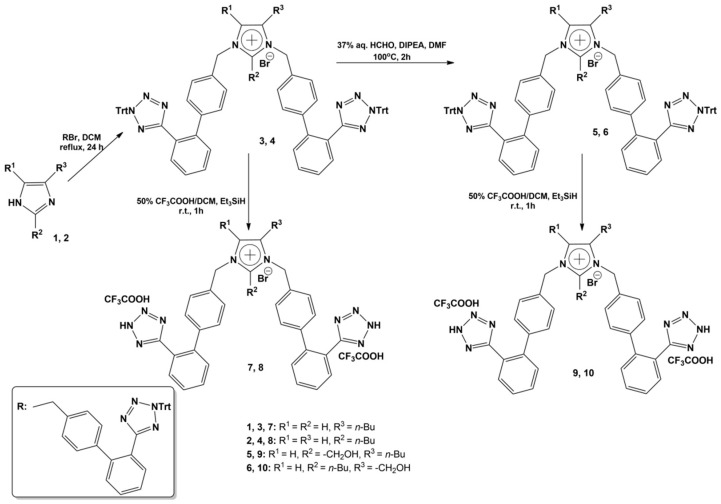
The scheme depicts the synthesis of imidazole-based bisartans bearing two symmetric biphenyl tetrazoles and butyl/CH_2_OH groups at positions 2 and 4, respectively.

There are two subclasses of sartans, imidazole and benzimidazole-based sartans. Examples of imidazole scaffold sartans are losartan, Exp3174 (carboxylic losartan), olmesartan, eprosartan, and all those sartans built on the imidazole ring. Examples of benzimidazole scaffold sartans are candesartan, telmisartan, Azilsartan, and those sartans built on benzimidazole moiety which fuses histidine and phenyl rings. Telmisartan was found to protect hypertensive patients infected by SARS-CoV-2 [57,58] and in general, the clinical trials advocate in favor of the beneficial effects of ARBs in blocking infection [26,27,31,59]. These studies showed that morbidity and mortality rate was lower in hypertensive patients infected by SARS-CoV-2 who were prescribed RAS and, in particular, ARBs inhibitors when compared to patients not taking these drugs [57,60,61,62]. Hypertensive patients not taking RAS antihypertensives are more vulnerable to developing serious complications of COVID-19. The imbalance in the renin–angiotensin system (excess of toxic angiotensin II against beneficial heptapeptides A(1-7), Alamantine) is responsible for hypertension and COVID-19. ARBs upregulate ACE2 which upgrades angiotensin II are proven to be beneficial against SARS-CoV-2 [63,64]. Scheme 1 shows the synthetic scheme for bisartan imidazole-based ligands **Co4**, **Co6**, **Co8**, and **Co10**.

### 3.7. Possible Target of Sartans as Revealed by Enzymatic Assays

In the current study, we selected to study the SPR binding to the human ACE2 protein of free sartans, not trityl protected, which were predicted to bind better to the ACE2-Spike RBD complex in computational studies, as depicted in Table 1 (best scoring pose bound to ACE2-Spike RBD complex). In particular, we used the bisartan BV6 (containing two tetrazoles), the sartan losartan (containing one tetrazole and one hydroxyl), and losartan carboxylic acid, Exp3174 (containing one tetrazole and one carboxyl), for the ACE2 study. The results presented showed an efficient binding of the three sartans. The order of binding BV6 > Losartan > Losartan carboxylic (Exp3174) suggests the superiority of tetrazolate compared to hydroxylate and carboxylate in binding to ACE2 in line with our previous studies, where BV6 was the best binder in computational studies (Ridgway et al. [22]). On the other hand, both sartans were found to inhibit the enzymatic activity of a recombinant soluble ACE2 only slightly (less than 10%) at a concentration of 100 micromolar, which suggests that sartans bind to amino acids at an area remote from the catalytic ACE2 Zn^2+^ center, not directly to the active site. Taken together, the binding and enzymatic studies suggest that the specific sartans do not selectively target the active site of the ACE2 enzyme but rather its interaction domain with the RBD of the viral S1 protein. 

### 3.8. Clinical Perspectives of Bisartans as COVID-19 Antivirals

The recent announcement of the Pfizer and Moderna antivirals, Paxlovid and Molnupiravir, triggered ongoing research for new antivirals against COVID-19 based on the action of their mechanism [11,12,13,14,15,16]. These drugs use different mechanisms of action to exert their antiviral activities against SARS-CoV-2 spike protein, first by blocking 3CLpro and second by interfering with RNA-dependent RNA polymerase, resulting in transcription error accumulation. Paxlovid’s active ingredient nirmatrelvir (PF-07321332) through the warhead nitrile group forming a covalent bond with thiol of the catalytic dyad Cys145- His41 of 3CLpro inactivates the catalytic site and blocks cleavage of the spike protein, thus preventing infection [10,14,65]. Our in silico studies showed that bisartans block the spike cleavage as arginine blockers through the three cell entries (ACE2, furin, 3CLpro) rendering them promising drugs for treating COVID-19 [21,22].

## 4. Materials and Methods

### 4.1. In Silico Methodology and Ligand Preparation

All compounds were sketched in the Chem3D 15.0 module of ChemOffice 15.0 and converted into SMILES [22]. This was accomplished using Maestro MacroModel 10.8 ( Schroedinger, LLC, New York, NY, USA) where all hydrogens were added and molecules were subjected to complete structure minimization. For the minimization, water was chosen as a solvent, OPLS3 [66] force field, and the algorithm Polak–Ribiere (PRCG, convergence value 0.01 kcal mol^−1^ Å^−1^) were used. The pH of the in silico studies was set to 7.4 based on the pH of human blood, which made our environment slightly basic. As a result, the protons of the tetrazole rings as well as the protons of the carboxylic acids are not present. The resulting files were saved in pdb format [67]. In AutoDock 4.0, flexible torsions were assigned allowing 100 conformations [68,69] and the acyclic dihedral angles were allowed to rotate freely. The files were then saved in the pdbqt file format for further analysis [67].

### 4.2. Molecular Docking

Molecular simulation studies of our compounds into the protein targets were carried out using the open-source program Autodock 4.0 included in Auto-Dock Tools 1.5.6. For this study, the crystal structures of the proteins were extracted by the Protein Data Bank. These results were compared to candesartan, a known ARB, from our previous study, as well as valsartan, olmesartan, and eprosartan [63]. The X-ray crystal structures of the spike receptor domain complexed with ACE2 (PDB ID: 6LZG) were downloaded from the RCSB PDB (Protein Data Bank) database [70,71]. Based on the literature, which was confirmed by blind docking, the S protein grid, surrounding Asn A 33, His A 34, Glu A 37, Asp A 38, Lys A 353, Ala A 387, Gln A 388, Pro A 389, Phe A 390, Arg A 393, Lys B 417, Tyr B 453, Tyr B 495, Gly B 496, Phe B 497, Ser B 494, and Tyr B 505, the critical interface residues of S protein-ACE-2 [72,73,74].

The best-docked poses, with both lower binding energies and stronger interaction patterns, were derived from the docking results and were visualized with PyMOL and the protein–ligand interaction profiler (PLIP; retrieved from https://pymol.sourceforge.net/overview/index.htm (accessed on 30 October 2022), PyMOL version 2.0, Schrödinger, Inc., New York, NY, USA) [75]. The PLIP was also used to determine the interactions between the ligands and the proteins [76]. The X-ray crystal structures of the selected SARS-CoV-2 proteins were downloaded from Protein Data Bank as pdb files, and Open Babel software was used to convert the protein files into pdbqt types. The co-crystallized ligands were removed; thus, 2-acetamido-2-deoxy-beta-D-glucopyranose of spike protein (PDB ID: 6LZG) [77]. The protein structures were refined for heteroatoms and water molecules to demarcate the active sites of the proteins. The hydrogen atoms and the nonpolar hydrogens were merged, and Gastgeiger and Kollman charges were added. In each receptor, the grid was set around its active site for site-specific docking to be performed [78]. The Lamarckian genetic algorithm (GA), which uses the AMBER force field to run the docking between the receptor and the ligand, was used for docking in combination with the grid-based energy evaluation method with the default parameters (GA: 5,000,000—energy evaluations and 175—population size). The program was run for a total number of 100 genetic algorithm runs. When using Autodock 4.0, the stability of the ligand/protein complex, which shows how efficiently can the ligand binds to the protein, is depicted with low energy. The lower the energy, the more efficient the ligand binds, which is depicted by the increased number of hydrogen bonds, non-covalent Van Der Waals, and hydrophobic interactions.

### 4.3. Docking Parameters

For in silico SARS-CoV-2 protein experiments with the selected ligands, the active site of each receptor was targeted. For the complex spike protein, with ACE2 as its predominant receptor (PDB ID: 6LZG) [77], the grid box was set with a spacing of 0.420 Å and dimensions of 77 Å × 80 Å × 100 Å, centering around residues mentioned above. In silico molecular docking analysis was performed with Autodock 4.0 [67]. The ligands produced pdbqt files, which were converted to pdb, using Open Babel to be compatible with the visualization platforms. Then, the pdb files were visualized and analyzed by PyMOL and PLIP [75,79].

### 4.4. Organic Synthesis of Bisartans

Starting materials were purchased by Aldrich (Patras, Greece) and were used as received. The 1H-NMR and 13C-NMR spectra were recorded on a Bruker Avance DPX spectrometer at 400.13 MHz and 161.76 MHz, respectively. Chemical shifts are given in δ values (ppm) using tetramethylsilane as the internal standard and coupling constants (J) are given in Hertz (Hz). HPLC analysis was performed on an Alliance Waters 2695 equipped with a Waters 2996 Photodiode Array Detector UV-Vis, using the XBridge Waters C18 column (4.6 × 150 mm, 3.5 μm) as stationary phase and a gradient of H_2_O/CH_3_CN, both containing 0.08% TFA as mobile phase. Electrospray-ionization mass spectra (ESI-MS) were obtained on a UPLC (ultra-performance liquid chromatography) equipped with SQ detector AcquityTM by Waters. Analytical TLC was performed on silica gel 60 F254 plates (Merck, Darmstadt, Germany) and visualized by UV irradiation. Silica gel 60N (particle size 0.04–0.063 mm) was used for flash column chromatography.

General procedure for the bis-alkylations: A solution of 4(5)-butylimidazole (**1**) or 2-butylimidazole (**2**) (0.8 mmol, 0.1 g) and 5-(4′-(bromomethyl)biphenyl-2-yl)-2-trityl-2H-tetrazole (1.69 mmol, 0,94 g) in 50 mL of dichloromethane (DCM) was refluxed for 24 h and the reaction was monitored by RP-HPLC (70% CH_3_CN in H_2_O to 100% CH_3_CN, in 30 min). The reaction mixture was diluted with 100 mL of dichloromethane and the organic solution was washed with 50 mL aq. KOH 1N, water (2 × 50 mL), and brine (50 mL). The organic layer was dried with over anhydrous Na_2_SO_4_, filtered and dichloromethane was evaporated in vacuo. In the oily residue, ethyl acetate (20 mL) and then, diethylether (100 mL) were added and a solid was precipitated. After filtration, the desired product (**3**) or (**4**) was afforded, in high yield and enough purity, in order to be used as it was for the next steps. Data for compound (**3**): Pale yellow solid, 0.73 g (78% yield), 95% purity (RP-HPLC). RP-HPLC (70% CH_3_CN in H_2_O to 100% CH_3_CN, in 30 min, t_R_: 16.71 min). R_f_ = 0.69 (90:10 CHCl_3_:MeOH). ESI-MS (M+H^+^): 1077.82, 1078.25. Data for compound (**4**): White solid, 0.75 g (80% yield), 95% purity (RP-HPLC). RP-HPLC (70% CH_3_CN in H_2_O to 100% CH_3_CN, in 30 min, t_R_: 14.93 min). R_f_ = 0.53 (90:10 CHCl_3_:MeOH). ESI-MS (M+H^+^): 1077.77, 1078.66. General procedure for the formylations: To a screw-capped glass tube, compounds (**3**) or (**4**) (0.26 mmol, 0.3 g), diisopropylethylamine (DIPEA) (1.30 mmol, 0.22 mL), 37% *w*/*w* formalin solution (2.60 mmol, 0.19 mL), and DMF (1 mL) were added and the mixture was heated at 100 °C for 2 h. Completion of the reaction was observed by RP-HPLC (70% CH_3_CN in H_2_O to 100% CH_3_CN, in 30 min) and the mixture was diluted with chloroform (200 mL). The organic layer was separated and washed with 10% *w*/*v* aq. citric acid (50 mL), water (50 mL), and brine (50 mL), dried with over anhydrous Na_2_SO_4_, filtered and chloroform was removed by vacuum evaporation. Diethylether (100 mL) was added to the oily residue and a white solid was precipitated, and, after filtration, the desired product (**5**) or (**6**) was afforded, in high yield and purity, and was used as it is to the deprotection reaction. Data for compound (**5**): White solid, 0.30 g (97% yield), 95% purity (RP-HPLC). RP-HPLC (70% CH_3_CN in H_2_O to 100% CH_3_CN, in 30 min, t_R_: 15.32 min). R_f_ = 0.60 (90:10 CHCl_3_:MeOH).ESI-MS (M+H^+^): 1107.59, 1108.28. Data for compound (**6**): White solid, 0.28 g (91% yield), 95% purity (RP-HPLC). RP-HPLC (70% CH_3_CN in H_2_O to 100% CH_3_CN, in 30 min, t_R_: 14.21 min). R_f_ = 0.28 (90:10 CHCl_3_:MeOH). ESI-MS (M+H^+^): 1107.81, 1108.80.

General procedure for the triphenylmethyl group removal: A quantity of a tetrazole Trt-protected bis-alkylated solid compound was dissolved in a 50% *v*/*v* trifluoroacetic acid (TFA) in dichloromethane solution and a few drops of triethylsilane (TES) were added, until decoloration of the mixture was observed. The resulting solution was stirred for 1 h at ambient temperature and then, was evaporated in vacuo. After diethylether addition, a solid was precipitated, which was obtained by filtration as an amorphous solid, in the form of the TFA salt of the respective protected compound. Data for compound (**7**): By deprotection of 0.2 g (0.17 mmol) of compound (**3**): White amorphous solid, 0.14 g (90% yield). RP-HPLC (20% CH_3_CN in H_2_O to 100% CH_3_CN, in 30 min, t_R_: 12.15 min). R_f_ = 0.36 (70:30 CHCl_3_:MeOH). ESI-MS (M+H^+^): 593.21. 1H-NMR (CD_3_OD): δ 9.02 (s, 1H), 7.70–7.67 (m, 4H), 7.61–7.54 (m, 4H), 7.46 (s, 1H), 7.36 (d, 2H, J = 8 Hz), 7.24–7.18 (m, 6H), 5.40 (s, 2H), 5.38 (s, 2H), 1.56 (quint., 2H, J = 7.2 Hz), 1.38 (q, 2H, J = 7.2 Hz), 1.17 (t, 2H, J = 7.2 Hz), 0.92 (t, 3H, J = 7.2 Hz). Anal. Calcd for C_35_H_33_N_10_Br·2CF_3_COOH (%): C: 51.95; H: 3.91; N: 15.53. Found (%): C: 52.00; H: 3.88; N: 15.99. Data for compound (**8**): By deprotection of 0.2 g (0.17 mmol) of compound (**4**): White amorphous solid, 0.13 g (84% yield). RP-HPLC (20% CH_3_CN in H_2_O to 100% CH_3_CN, in 30 min, t_R_: 11.25 min). R_f_ = 0.32 (70:30 CHCl_3_:MeOH). ESI-MS (M+H^+^): 593.33. 1H-ΝΜR (CD_3_OD): δ 7.68–7.65 (m, 4H), 7.59–7.52 (m, 5H), 7.27–7.18 (m, 9H), 5.44 (s, 4H), 3.03 (t, 2H, J = 8 Hz), 1.37–1.28 (m, 4H), 0.84 (t, 3H, J = 7.2 Hz). Anal: Calcd for C_35_H_33_N_10_Br·2CF_3_COOH (%): C: 51.95; H: 3.91; N: 15.53. Found (%): C: 52.08; H: 3.90; N: 15.89. Data for compound (**9**): By deprotection of 0.2 g (0.17 mmol) of compound (5): White amorphous solid, 0.15 g (95% yield). RP-HPLC (20% CH_3_CN in H_2_O to 100% CH_3_CN, in 30 min, t_R_: 12,10 min). R_f_ = 0.33 (70:30 CHCl_3_:MeOH). ESI-MS (M+H^+^): 623.18. 1H-ΝΜR (CD_3_OD): δ 6.90–6.86 (m, 4H), 6.81–6.74 (m, 4H), 6.63 (s, 1H), 6.53 (d, 2H, J = 8 Hz), 6.42–6.30 (m, 6H), 4.75 (s, 2H), 4.71 (s, 2H), 4.12 (s, 2H), 1.72 (t, 2H, J = 8 Hz), 0.72 (quint., 2H, J = 7.2 Hz), 0.55 (q, 2H, J = 7.2 Hz), 0.09 (t, 3H, J = 7.2 Hz). Anal. Calcd for C_36_H_35_N_10_Br·2CF_3_COOH (%): C: 51.57; H: 4.00; N: 15.03. Found (%): C: 52.06; H: 3.95; N: 15.17. Data for compound (**10**): By deprotection of 0.2 g (0.17 mmol) of compound (**6**): White amorphous solid, 0.15 g (95% yield). RP-HPLC (20% CH_3_CN in H_2_O to 100% CH_3_CN, in 30 min, t_R_: 11.65 min). R_f_ = 0.29 (70:30 CHCl_3_:MeOH).ESI-MS (M+H^+^): 623.25. 1H-ΝΜR (CD_3_OD): δ 7.73 (d, 2H, J = 7.2 Hz), 7.49–7.44 (m, 2H), 7.39–7.35 (m, 7H), 7.28–7.25 (m, 4H), 7.21 (d, 2H, J = 8 Hz), 5.63 (s, 2H), 5.45 (s, 2H), 4.62 (s, 2H), 3.01 (t, 2H, J = 8 Hz), 1.27–1.17 (m, 2H), 1.14–1.06 (m, 2H), 0.70 (t, 3H, J = 7.2 Hz). Anal. Calcd for C_36_H_35_N_10_Br·2CF_3_COOH (%): C: 51.57; H: 4.00; N: 15.03. Found (%): C: 51.88; H: 3.93; N: 15.11. NMR data: 1H-ΝΜR (DMSO-d6): δ 8.10 (s, 1H), 7.76 (s, 1H), 7.65 (s, 2H), 7.57–7.51 (m, 3H), 7.24–7.20 (m, 2H), 7.11–7.07 (m, 2H), 6.38 (d, 1H, J = 16.0 Hz), 5.24 (s, 2H), 5.04 (s, 2H), 2.16 (s, 3H). Anal. Calcd for C_25_H_20_N_6_O_5_·CF_3_COOH (%): C: 54.18; H: 3.54; N: 14.04. Found (%): C: 54.08; H: 3.41; N: 14.17.

### 4.5. ACE2 Protein Purification

The human ACE2 protein, tagged with His-tag and Strep-tag, was overproduced in Sf 9 insect cells. For purification, 107 cells were used. The ACE2 tagged protein was purified with the usage of Strep-Tactin Superflow Plus resin (Qiagen, Hilden, Germany), according to the manufacturer’s protocol. Cells were lysed in the NP buffer (50 mM NaH2PO4 pH 8, 300 mM NaCl, 10% (*v*/*v*) glycerol, 1% (*v*/*v*) Triton X-100) containing proteases inhibitors (Pierce™ Protease Inhibitor Mini Tablets). The clear cellular lysate was incubated with the resin for 1 h with rotation. Next, the resin was washed with NP buffer and ACE2 protein was eluted with NP buffer containing 10 mM biotin hydrazide. 

### 4.6. Surface Plasmon Resonance Analysis

SPR experiments were performed with Biacore T200, (Cytiva, Malborough, MA, USA) equipment [80]. The purified ACE2-tagged protein was covalently immobilized on a CM5 Sensor Chip (Cytiva, Malborough, MA, USA) via the primary amine group, in a 10 mM sodium acetate buffer, pH 4.5, as described by Zhu and co-workers. The protein was immobilized to a response level of 7700 RU. Experiments were run at 25 °C, and the running buffer was PBS-P (Cytiva, Malborough, MA, USA) with 2% DMSO. The sartans were prepared running buffer in a series of concentrations from 0.6 to 180 µM and flowed over the immobilized ACE2 protein at a flow rate of 30 μL/min, contact time of 120 s, and dissociation time of 300 s. The sensor chip surface was regenerated using 1.5 M NaCl and 10 mM glycine, pH 1.5. After each analysis, an additional wash with 50% DMSO solution was performed. The results are presented as sensorgrams obtained after subtracting the background response signal from a reference flow cell and a control experiment with buffer injection. For ACE2–Losartan interaction, a kinetic constant was calculated with Biacore T200 Evaluation Software using the 1:1 binding model.

## 5. Conclusions

The present in silico study revealed the significant role of the tetrazole of sartans and bisartans for binding to the ACE2/Spike complex. The protection of warhead tetrazole with trityl group reduced the affinity of sartans to the complex, confirming the importance of negative tetrazolate for binding. SPR binding analysis confirmed the interaction of tetrazole-containing sartans with ACE2. Imidazole and benzimidazole scaffold-based sartans with tetrazole and carboxylate, as warheads, are promising compounds for treating COVID-19 and are worthy of further investigation.

## Figures and Tables

**Table 1:** Interactions of amino acid residues of the main protease of SARS-CoV-2 (PDB ID: 6LZG) with the ligands using PLIP.

Structures	Ligands		Best Scoring Pose Bound to ACE2-Spike RBD Complex (kcal/mol)
**1**	**Co1**		−4.73
**2**	**Co2**		−4.87
**3**	**Co3**	trityl protected	−9.4
**4**	**Co4**	free	−10.68
**5**	**Co5**	trityl protected	−9.71
**6**	**Co6**	free	−12.1
**7**	**Co7**	trityl protected	−6.81
**8**	**Co8**	free	−10.12
**9**	**Co9**	trityl protected	−6.8
**10**	**Co10**	free	−11.34
**11**	**valsartan**		−8.53
**12**	**olmesartan**		−7.88
**13**	**eprosartan**		−6.98
**14**	**losartan**		−7.74

**Table 2:** The average values of binding affinity for the complexes of sartans with COVID-19 S-Protein.

Type of Interactions	Amino Acids of S-RBD Involved and Distance of Interactions (Å)			
	Valsartan	Olmesartan	Eprosartan	Losartan
Hydrogen Bonds	403B Arg 3.03 406B Glu 3.06	99A Ala 2.11 390A Phe 2.52 393A Arg 2.17	69A Trp 2.14	393A Arg 2.12 393A Arg 2.43 394A Asn 2.86
Hydrophobic Interactions	33A Asn 3.66 34A His 3.56 37A Glu 3.66 38A Asp 3.67 505B Tyr 3.20	73A Leu 3.11 99A Ala 3.79 350A Asp 3.67 390A Phe 3.59 391A Leu 3.34 394A Asn 3.61	0A Phe 3.23 69A Trp 3.28 73A Leu 3.25 391A Leu 3.34 393A Arg 3.18 394A Asn 3.85	40A Phe 3.90 73A Leu 3.48 100A Leu 3.55 350A Asp 3.33 390A Phe 3.33 390A Phe 3.83 391A Leu 3.30
π–Cation Interactions	353A Lys 3.89 403B Arg 4.26	-	-	-
Salt Bridges	403B Arg 3.35	562A Lys 2.68	562A Lys 2.74	562A Lys 3.19

**Table 3:** The average values of binding affinity for the complexes of our generated small compounds **Co1**, **Co2** and the bigger, trt-protected compounds, **Co3**, **Co5**, **Co7**, and **Co9** with COVID-19 S-Protein.

Type of Interactions	Amino Acids of S-RBD Involved and Distance of Interactions (Å)					
	Co1	Co2	Co3	Co5	Co7	Co9
Hydrogen Bonds	34A His 2.13 38A Asp 3.57 353A Lys 3.04 494B Ser 2.05 496B Gly 2.72	353A Lys 2.20 453B Tyr 2.11 496B Gly 2.28	33A Asn 3.06	403B Arg 3.11	33A Asn 3.76 393A Arg 3.51 403B Arg 2.67 405B Asp 3.18	26A Lys 1.87 33A Asn 3.64 386A Ala 3.02 393A Arg 2.97 505B Tyr 3.26
Hydrophobic Interactions	497B Phe 3.41 497B Phe 3.68 505B Tyr 3.52	495B Tyr 3.10 497B Phe 3.42 497B Phe 3.57 505B Tyr 3.57	33A Asn 3.75 34A His 3.87 37A Glu 3.09 321A Pro 3.84 324A Thr 3.59 356A Phe 3.24 356A Phe 4.00 383A Met 3.75 386A Ala 3.04 387A Ala 3.27 389A Pro 3.26 417B Lys 3.39 505B Tyr 3.34	23A Glu 3.77 26A Lys 2.95 26A Lys 3.16 29A Leu 3.72 29A Leu 3.23 30A Asp 3.78 37A Glu 3.93 37A Glu 3.94 93A Val 3.20 405B Asp 3.63 406B Glu 3.61 420B Asp 3.76 456B Phe 3.98 505B Tyr 3.91	30A Asp 3.98 33A Asn 3.65 34A His 3.84 37A Glu 3.18 386A Ala 3.23 387A Ala 3.78 387A Ala 3.63 389A Pro 3.87 389A Pro 3.62 417B Lys 3.42 555A Phe 3.75	26A Lys 3.61 27A Thr 3.87 30A Asp 3.70 33A Asn 3.80 34A His 3.96 37A Glu 3.63 387A Ala 3.86 405B Asp 3.52 406B Glu 3.95 417B Lys 3.90 417B Lys 3.50 505B Tyr 3.81
π–Stacking			34A His 4.06	34A His 3.98 421B Tyr 5.30	34A His 3.90	
π–Cation Interactions	353A Lys 3.86		34A His 5.61 403B Arg 3.89 403B Arg 5.91	403B Arg 5.21 417B Lys 5.49	408B Arg 5.55	417B Lys 5.39

**Table 4:** The average values of binding affinity for the complexes of our generated compounds **Co4**, **Co6**, **Co8**, and **Co10**, (which do not contain the trt-group like their **Co1**–**Co3**, **Co5**, **Co7**, and **Co9** counterparts) with COVID-19 S-Protein. The average values of binding affinity for the complexes of our generated compounds **Co4**, **Co6**, **Co8**, and **Co10**, (which do not contain the trt-group like their **Co1**–**Co3**, **Co5**, **Co7**, and **Co9** counterparts) with COVID-19 S-Protein.

Type of Interactions	Amino Acids of S-RBD Involved and Distance of Interactions (Å)			
	Co4	Co6	Co8	Co10
Hydrogen Bonds	44A Ser 1.92 44A Ser 3.19 350A Asp 2.45 394A Asn 2.74 562A Lys 2.13	37A Glu 3.16 353A Lys 3.07 353A Lys 3.26 354A Gly 2.85 403B Arg 3.01 496B Gly 2.23 505B Tyr 2.73 505B Tyr 1.84	34A His 3.05 388A Gln 1.99 393A Arg 2.14 403B Arg 2.64 406B Glu 2.49 559A Arg 2.32	44A Ser 3.58 350A Asp 2.49 350A Asp 2.14 390A Phe 2.22 393A ARg 2.82 394A Asn 2.79 562A Lys 2.28
Hydrophobic Interactions	73A Leu 3.28 347A Thr 3.95 349A Trp 3.20 349A Trp 3.89 350A Asp 3.25 391A Leu 3.17	34A His 3.52 38A Asp 3.52 356A Phe 3.53 386A Ala 3.41 405B Asp 3.71 406B Glu 3.74 453B Tyr 3.47 493B Gln 3.99 505B Tyr 3.40	33A Asn 3.38 37A Glu 3.35 389A Pro 3.45	40A Phe 3.22 40A Phe 3.56 99A Ala 3.44 347A Thr 3.65 349A Trp 3.70 350A Asp 3.18 391A Leu 3.13
π–Stacking	390A Phe 4.00	495B Tyr 5.21 505B Tyr 4.50		390A Phe 4.33
π–Cation Interactions		34A His 4.65 353A Lys 3.23		

## Data Availability

Data sharing is not applicable to this article.

## Abbreviations

COVID-19: Coronavirus disease; SARS-CoV-2, Severe Acute Respiratory Syndrome Corona Virus; ACE2, Angiotensin Converting Enzyme II; 3CLpro, 3-chymotrypsin like protease; AT1R, angiotensin II T1receptor; ARBs, Angiotensin Receptor Blockers; RBD, Receptor Binding Domain; NEP, Neprilisin; Cande, Candesartan; Telmi, Telmisartan; Olme, Olmesartan; Azil, Azilsartan; Lo, Losartan; Irbe, Irbesartan; DIZE, deminazene aceturate; Epro, Eprosartan; EXP3174, losartan carboxylic acid; Val, Valsartan; BisA, 4-Butyl-N,N0-bis{[[20-(2H-tetrazol-5-yl)]biphenyl-4-yl]methyl}imidazolium bromide; BisB, 4-Butyl-2-hydroxymethyl-N,N0-bis{[20-(2H-tetrazol-5-yl)biphenyl-4-yl]methyl}imidazolium bromide; BisC, 2-Butyl-4-chloro-5-hydroxymethyl-N,N0-bis{[20-(2H-tetrazol-5-yl)biphenyl-4-yl]methyl}imidazolium bromide; BisD, 2-Butyl-N,N0-bis{[20-(2H-tetrazol-5-yl)biphenyl-4-yl]methyl}imidazolium bromide.
